# Infrared Spectroscopy with a Fiber-Coupled Quantum Cascade Laser for Attenuated Total Reflection Measurements Towards Biomedical Applications

**DOI:** 10.3390/s19235130

**Published:** 2019-11-23

**Authors:** Ine L. Jernelv, Karina Strøm, Dag Roar Hjelme, Astrid Aksnes

**Affiliations:** Department of Electronic Systems, Norwegian University of Science and Technology (NTNU), O.S. Bragstads plass 2A, 7491 Trondheim, Norway; strmkarina@gmail.com (K.S.); dag.hjelme@ntnu.no (D.R.H.); astrid.aksnes@ntnu.no (A.A.)

**Keywords:** biomedical spectroscopy, quantum cascade lasers, mid-infrared, glucose

## Abstract

The development of rapid and accurate biomedical laser spectroscopy systems in the mid-infrared has been enabled by the commercial availability of external-cavity quantum cascade lasers (EC-QCLs). EC-QCLs are a preferable alternative to benchtop instruments such as Fourier transform infrared spectrometers for sensor development as they are small and have high spectral power density. They also allow for the investigation of multiple analytes due to their broad tuneability and through the use of multivariate analysis. This article presents an in vitro investigation with two fiber-coupled measurement setups based on attenuated total reflection spectroscopy and direct transmission spectroscopy for sensing. A pulsed EC-QCL (1200–900 cm${}^{-1}$) was used for measurements of glucose and albumin in aqueous solutions, with lactate and urea as interferents. This analyte composition was chosen as an example of a complex aqueous solution with relevance for biomedical sensors. Glucose concentrations were determined in both setup types with root-mean-square error of cross-validation (RMSECV) of less than 20 mg/dL using partial least-squares (PLS) regression. These results demonstrate accurate analyte measurements, and are promising for further development of fiber-coupled, miniaturised in vivo sensors based on mid-infrared spectroscopy.

## 1. Introduction

Sensor development in the mid-infrared (MIR) region is interesting for many applications, with research ranging from sensor chips for gas detection to biomedical applications [1,2]. Biomedical applications are particularly important due to rising costs associated with healthcare, and a need for rapid, reagent-free, and non-destructive measurement techniques. Sensing in the MIR wavelength range, typically defined as 2.5–25 μm (4000–400 cm${}^{-1}$), has advantages over several other optical measurement methods [3]. For example, MIR spectroscopy measures the absorption of fundamental molecular vibrations, which gives relatively sharp and strong absorption bands.

MIR spectroscopy has been a standard laboratory technique for decades through the use of e.g., Fourier-transform interferometer (FTIR) spectrometers, but these benchtop instruments have several limitations regarding on-demand and portable sensing. FTIR spectrometers are traditionally bulky instruments with free-space optics and require relatively large sampling volumes. Additionally, FTIR spectrometers commonly use thermal emitters such as SiC globars, which provide a low overall spectral power density in the MIR wavelength range. Consequently, these spectrometers have had limited sensitivity for measurements of biomedical samples, as they usually contain water which strongly absorbs in the MIR range. Other alternatives, such as CO${}_{2}$ lasers or lead–salt lasers, can provide higher spectral power density. However, these lasers have seen limited use for biomedical applications due to narrow wavelength ranges, and a need for cryogenic operating temperatures in the case of lead–salt lasers [4,5].

The introduction of broadly tuneable quantum cascade lasers (QCLs) has sparked new research interest within this field. The main advantage of QCLs is their high spectral emission power, which can reach a factor of 10${}^{4}$ higher than that of the thermal sources used in FTIR spectrometers. Additionally, QCLs are small, can be made tuneable over several hundred wavenumbers, and can be operated with thermoelectric cooling [6]. This makes QCLs well-suited for sensor development. Several resonator designs are routinely used in QCLs [2]. The simplest design is the Fabry-Pérot (FP) resonator, which is made by cleaving the ends of the gain chip. FP-QCLs have multimode emission, and can be tuned a few wavenumbers by changing the temperature or current over the chip. Singlemode emission from a QCL can be achieved by making a Bragg grating on the laser chip, commonly referred to as distributed feedback (DFB) technology. The tuning range of a DFB-QCL is approximately 5 cm${}^{-1}$, which makes this resonator design more suitable for gas spectroscopy, although multiple DFB-QCLs can be combined for a wider spectral range. The use of an external cavity (EC) provides a broader tuning range up to several hundred cm${}^{-1}$, with emission in either continuous wave or pulsed mode. EC-QCLs can therefore be used for detection of several analytes through multivariate analysis.

QCLs are particularly advantageous for biomedical applications. For benchtop uses the QCLs are compact, they have high spectral power density which gives a high signal-to-noise ratio (SNR) even in aqueous samples, and can reach measurement times down to a few seconds. For personal and portable uses, QCLs have a large potential for miniaturised sensors, especially with single-wavelength laser chips. Some miniaturised MIR sensors with thermal sources have been made, many with MOEMS-based (micro-opto-electromechanical systems) technology [7,8], but these are still most suited for gas sensing. Today, portable sensors that are used clinically instead typically rely on enzymatic reactions. As an example, glucose sensors for monitoring diabetes are the most common type of portable biomedical sensor [9]. Monitoring the glucose level is essential for diabetic patients, and is done either with fingerprick measurements of blood or with continuous glucose monitoring (CGM) devices worn on the body [10]. In both cases the measurements are facilitated by an enzymatic reaction, which for the CGM devices limits the sensor lifetime to less than two weeks due to sensor and reagent degradation [11,12]. Optical methods such as MIR spectroscopy can circumvent some of these difficulties as the measurements are reagent-free. However, other complications exist for optical methods, including differentiation of overlapping spectral bands and obtaining accurate measurements of low analyte concentrations [13].

QCLs have been employed by several research groups in setups for glucose measurements, aimed at use in portable sensors or larger sensors for intensive care, mainly using variations of transmission or attenuated total reflection (ATR) spectroscopy [14,15,16,17,18,19,20]. Promising initial results were also shown for in vivo glucose monitoring in interstitial fluid with a QCL, albeit with short-term measurements using a single-wavelength laser [21]. Other variants such as measurements of the photoacoustic signal or backreflected light from skin have also been investigated [22,23,24,25,26], but non-invasive sensing through skin is challenging in the MIR range due to strong water absorption.

Transmission and ATR measurements are both done through absorption spectroscopy, but ATR spectrocopy uses the evanescent field decaying out from waveguides or prisms where radiation has undergone total internal reflection (TIR). Different materials are used to enable evanescent sensing in ATR spectroscopy. ZnS offers a good compromise as a prism material in biomedical applications as it is cheap, has a high refractive index (approx. 2.2 at 10 μm), and is non-toxic. The Matsuura group has several publications on fiber-coupled ATR spectroscopy aimed toward non-invasive measurements of the inner lip mucosa [16,27,28]. They have used either an FTIR spectrometer as a source, which has a low power density, or 2–3 single-wavelength QCLs, which suffers from drift between the lasers. Another recent study showed detection of glucose in saliva in an EC-QCL ATR setup, but the water matrix was evaporated from the sample before measurements and the setup was in free-space [18]. Using a fiber-coupled system would have several advantages, as it simplifies further sensor development in regard to reducing sensor dimensions and portable sensing. A combination of ATR spectroscopy and fiber-coupling could be used in a probe design suitable for a portable sensor. This configuration could potentially be employed for minimally invasive sensing right under the skin. So far, studies of fiber-coupled ATR setups with EC-QCL sources have not been reported in detail, and a robust study of the achievable sensitivity in aqueous solutions is required.

In this article, we report on a fiber-coupled EC-QCL setup with sensing based on ATR spectroscopy with a ZnS prism. The ATR measurements are compared to transmission measurements, with transmission directly through a gap between two optical fibers. Comparing the ATR setup with a transmission setup will serve to validate the findings in the ATR setup. The sensing capabilities of these setups are tested by measuring aqueous solutions with glucose and albumin, as well as lactate and urea as interfering species. These analytes were chosen as an example system as they are present in biofluids, and glucose and proteins are especially relevant for biomedical sensors.

## 2. Materials and Methods

### 2.1. Experimental Setup

**Figure 1:**
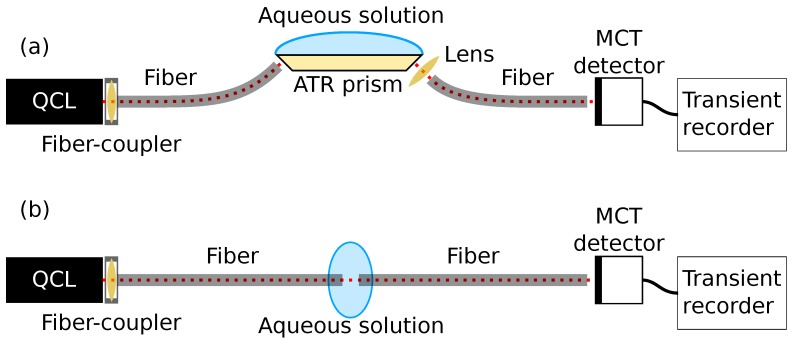
Overview of the two experimental setups, with sensing through (**a**) ATR spectroscopy and (**b**) transmission spectroscopy. For simplicity, the laser controller, the trigger line going from the laser controller to the transient recorder, and the control computer are not shown.

The setup (Figure 1) employed an external-cavity quantum cascade laser (EC-QCL, Hedgehog-UT, Daylight Solutions, San Diego, CA, USA) with a maximum tuning range between 1200 cm${}^{-1}$ and 900 cm${}^{-1}$ (8.33–11.1 μm). The EC-QCL was operated in pulsed mode with a pulse width of 500 ns and a repetition rate of 100 kHz, giving a duty cycle of 5%. The maximum average power for these settings was 22 mW. The laser head was thermoelectrically cooled to 19 ${}^{\circ }$C for all measurements. The laser head emitted radiation in a collimated beam which was 100:1 vertically polarised according to the specifications. A mercury-cadmium-telluride (MCT) detector (Vigo System, Ozarow Mazowiecki, Poland) with a 2 × 2 mm detector element was used to detect the mid-infrared radiation. This detector used a four-stage thermoelectric cooling system in order to operate at −75.2 ${}^{\circ }$C, and had a detectivity of 3 × 10${}^{9}$ cm Hz${}^{0.5}$ W${}^{-1}$.

The emitted beam was coupled into a silver halide fiber with an optical assembly (OptoKnowledge Systems, Los Angeles, CA, USA). This optical assembly was designed to minimise the coupling loss in the wavelength range for the laser, with a loss of <5%. In the ATR setup, these fibers were used to couple the radiation into and out of a ZnS prism (Sinoptix, Shanghai, China). The ZnS prism was a trapezoid with 45 degree facets for in- and out-coupling of radiation. The top facet of the prism was 24 × 6 mm${}^{2}$, and the prism height was 2.4 mm. The silver halide fiber (Art photonics, Berlin, Germany) used for in-coupling had a core size of 400 μm, while the out-coupling fiber had a core size of 600 μm, both with optical losses of 0.2–0.3 dB/m. Radiation exiting from the ZnS prism was focussed onto the out-coupling fiber using a ZnSe lens (Thorlabs, Newton, NJ, USA). In the transmission measurements, the fibers were aligned and a 165 μm gap between the fibers was used for sensing. The transmission pathlength was chosen based on a trade-off between analyte absorbance signal and the noise level, and was similar to the optimal pathlength found in other work [15].

Data acquisition was done with an analogue-to-digital converter card (M2p.5946-X4, 80 MS/s, 16 bit, Spectrum Instrumentation, Großhansdorf, Germany). The digitiser was controlled with a modified driver written in C++, and operated via a GUI front-end written in Python. A trigger signal between the laser controller and the digitiser card was used to start the data acquisition for each measurement. Spectra were acquired by operating the laser in scan-mode, where the laser was continuously tuned over a chosen wavelength range.

### 2.2. Data Processing

For each spectrum, a background measurement (I${}_{0}$) and a sample measurement (I) were recorded for the 1200–925 cm${}^{-1}$ tuning range. Demineralised water was used for the background measurements. Each background and sample measurement consisted of ten scans over the tuning range. Spectra were made by averaging over 255 laser pulses from the raw data, and then averaging over the 10 acquired scans from each measurement, for the purpose of noise reduction. Each scan took 1 s to acquire with a tuning speed of 275 cm${}^{-1}$/s, and consisted of approximately 100,000 pulses. Each spectrum was reduced to 390 data points after the spectral binning. From this, the absorbance spectra of the samples were calculated as A = −log(I/I${}_{0}$).

An alignment procedure was also performed on the spectra in order to correct for offset between scans and measurements. The scans were aligned to the first scan of each measurement by a chi-squared minimisation algorithm. Subsequently, each sample measurement was aligned to a background measurement.

Data analysis for prediction of analyte concentrations was done using a program developed in-house (Python). Partial least-squares (PLS) regression was used for prediction of glucose concentrations [29]. The data sets were standardised prior to PLSR by subtracting the mean and scaling to unit variance. Smoothing with a Savitzky–Golay filter was also applied as a pre-processing method [30]. The root-mean-square error of cross-validation (RMSECV) and the coefficient of determination (R${}^{2}$) were used to evaluate the prediction accuracy of the regression. RMSECV was calculated from either a leave-one-out cross-validation (LOOCV), leave-5-out cross-validation (L5OCV), or leave-one-dataset-out cross-validation (LDOCV).

We would like to note that RMSE-values are scale-dependent, which means that the RMSE will vary if different concentration ranges are used. As a result, RMSE-values are difficult to compare between studies if different concentration ranges are used. R${}^{2}$ has therefore been included as an alternative evaluation metric.

### 2.3. Sample Overview

Aqueous solutions were made by dissolving the analytes in a phosphate-buffered saline (PBS) solution. PBS is a buffer solution that has ion concentrations matching the human body and helps maintain pH, and can be made by dissolving PBS tablets (VWR) in water. Glucose (D-(+)-glucose, Sigma Aldrich), albumin (bovine serum albumin, VWR), lactate (sodium L-lactate, Sigma Aldrich), and urea (Sigma Aldrich) were added to solutions in varying concentrations. 25 unique solutions were made, with concentration ranges as shown in Table 1.

Analyte concentrations were designated to cover the entire design space, and were determined by a quadratic Scheffe model with A-optimality design. Optimal design allows for using fewer samples in the analysis while still maintaining robust concentration predictions [31]. Ten additional samples with glucose in demineralised water were also made for initial system characterisation, with glucose concentrations in the range 0–810 mg/dL.

ATR measurements were performed by placing the sample on the ZnS crystal, and the crystal was wiped clean with ethanol between each measurement. Transmission measurements were done by putting the sample in a gap between two fibers, and the gap was emptied and rinsed with ethanol between each measurement. For the two setup configurations, all samples were measured in four different series, with two series each on two different days.

## 3. Results and Analysis

### 3.1. Optical Propagation in ZnS Prism

**Figure 2:**
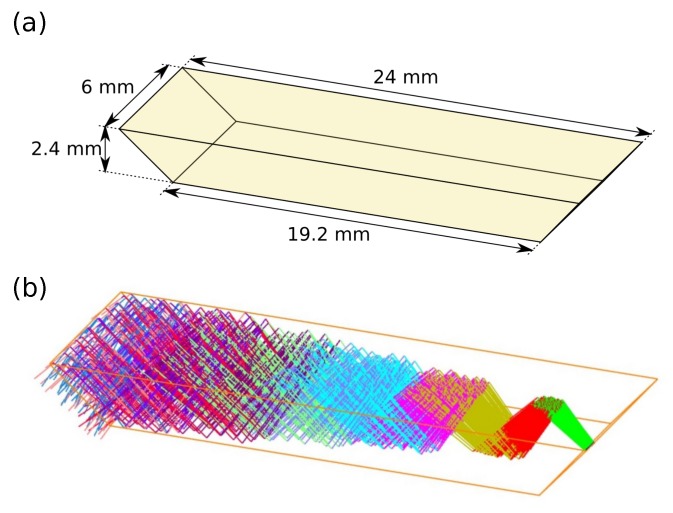
(**a**) Geometry of ZnS prism, and (**b**) ray-tracing simulation (Zemax OpticStudio) of trajectories of IR radiation in the prism.

Propagation of light in the ZnS ATR prism was simulated in Zemax OpticStudio using ray tracing, see Figure 2. The radiation source was modelled as a beam with an initial diameter of 400 μm, as this was the core size of the fiber. The divergence angle of this beam was set according to the numerical aperture (NA = 0.30) of the silver halide fiber. This created nine reflections in total as the beam propagated through the crystal, with five reflections on the top facet. The propagation length inside the prism is calculated to approximately 30.5 mm.

The beam diverges rapidly inside the prism due to the large initial size and the divergence angle from the optical fiber. Due to this divergence, the more radial parts of the beam will have a somewhat longer pathlength than the center, and will therefore reach the detector at a different time. For most of the radial parts of the beam, the largest pathlength difference is calculated to 0.25 mm.

The penetration depth for the evanescent field extending from the prism is between approximately 4 μm and 5.4 μm depending on the wavelength [32]. With 5 reflections this gives in a total interaction length of 20–27 μm.

### 3.2. Laser Intensity Variation

The pulsed operation of the QCL means that there is inherently some intensity variation between laser pulses. It is therefore common to average, or bin, over several laser pulses in order to reduce this variation.

**Figure 3:**
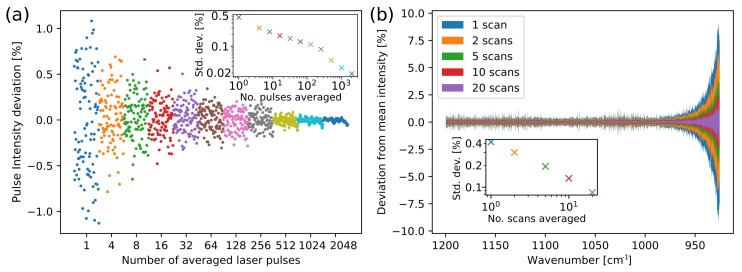
(**a**) Characterisation of the pulse-to-pulse intensity variation from the laser with averaging over pulses at a single wavelength, where the inset shows how the relative standard deviation decreases with pulse averaging. (**b**) Characterisation of scan-to-scan variation with averaging over multiple scans, where the inset shows the average relative standard deviation using the 1200–1000 cm${}^{-1}$ range.

In order to characterise pulse-to-pulse variation 100,000 laser pulses at 1190 cm${}^{-1}$ (8.4 μm) were recorded. Additionally, to investigate scan-to-scan variation 50 separate scans in the range 1200–925 cm${}^{-1}$ were recorded. Example results of averaging over pulses and scans are shown in Figure 3. These measurements were recorded in the fiber-coupled transmission setup, after transmitting the beam through a 165 μm layer of water.

Pulse averaging was tested from one to 2560 pulses, while scan averaging was tested from one to 20 scans. The relative standard deviation (RSD) decreases when averaging over more pulses, and when averaging over several scans. Averaging over scans in the transmission setup also reveals that the standard deviation becomes much larger for wavenumbers below approximately 970 cm${}^{-1}$ (longer than 10.3 μm), see Figure 3b. The increase in noise can be attributed to the stronger water absorption in this wavelength area. The result is not unexpected, and the water absorption should be taken into account when choosing pathlength. In our case, we maintained the long pathlength in the transmission setup in order to increase sensitivity, as none of the analytes were expected to have crucial information below 970 cm${}^{-1}$. This effect on the RSD is much smaller in the ATR setup, as the interaction length is much shorter.

Averaging over pulses is a trade-off between the reduction in noise, and measurement time or wavelength resolution. In this system, averaging over 10 scans and 255 pulses in each measurement gave an RSD of approx. 0.07%, which is equivalent to 0.0003 absorbance units. For the transmission measurements, this RSD was calculated for 1200–1000 cm${}^{-1}$, where the laser noise was dominant. Each scan takes 1 s to perform and contains 100,000 pulses, which gives a total measurement time of 10 s and a nominal resolution of 0.7 cm${}^{-1}$.

### 3.3. Glucose Spectra

**Figure 4:**
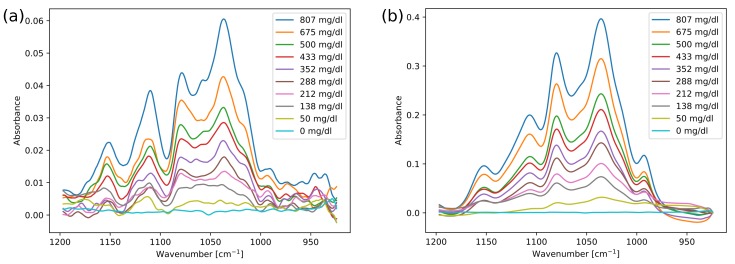
Absorbance spectra of different glucose concentrations for (**a**) measurements acquired in the ATR setup and (**b**) measurements acquired in the transmission setup.

Ten glucose solutions were measured in the ATR and transmission configurations in order to characterise the setups. Typical spectra from these measurements are shown in Figure 4, after being subjected to a Savitzky–Golay filter (width 9). These measurements were also repeated within the same day and on separate days, for a total of four measurement series in each setup.

As the setups are based on absorption spectroscopy, there are no major structural differences between the spectra, and the same glucose absorption peaks are present. There are some distortions in the ATR spectra, such as the lower dip around 1090 cm${}^{-1}$. Band distortions are known to occur in ATR spectroscopy, and are typically explained by anomalous dispersion (AD) in the refractive index of the measured sample [33]. Distortions caused by AD usually involve both intensity variations and frequency shifts, while the spectra in Figure 4a mainly display intensity variations. In addition, the spectral distortions are almost independent of glucose concentration, contrary to the concentration-dependent distortion expected from AD effects. This is in agreement with previous results, as e.g., Rowe et al. [34] found a change in refractive index of <0.01 in whole blood as compared to water. Band distortions are also found in other spectroscopy techniques that involve reflections, e.g., transflection spectroscopy [35]. It has been suggested that band variations in transflection measurements can be caused by reflections and interferences at interfaces [36], and a similar effect could also apply to ATR configurations. We therefore find it likely that the distortions occur due to reflections in optical components in the setup. The distortions in the ATR spectra did not affect the linearity of the glucose absorbance. However, the absorbance intensity in the ATR measurements is approximately 7 times lower than for the transmission measurements, which results in a lower overall SNR.

The main cause of the lower absorbance in the ATR measurements is the relatively shorter interaction length between the radiation and the sample in the ATR setup versus the transmission setup. The estimated interaction length of 20–27 μm in the prism agrees well with the seven-factor difference in absorbance. This also indicates that other causes for signal loss are relatively unimportant in our configuration, for example through material scattering in the prism and losses at the out-coupling facet of the prism.

Glucose concentrations can easily be predicted in these simple aqueous solutions. The prediction errors are summarised in Table 2. Using PLS regression and LOOCV, the RMSECV for glucose was found to be 8.5 mg/dL for the transmission measurements, and 10.2 mg/dL for the ATR measurements. These RMSECV values were found using 3 latent variables (LVs). For LDOCV, where datasets from four different measurements series were used in the cross-validation, the RMSECV values were slightly higher at 12.8 mg/dL for the ATR setup and 9.7 mg/dL for the transmission setup.

### 3.4. Sample Analysis

In more realistic sensing scenarios, it is important that the sensor is selective. It is also advantageous if the sensor can be used for more than one analyte, e.g., for management and tracking progression of several disease markers simultaneously. Therefore, more complex solutions with glucose, albumin, lactate, and urea were measured in the ATR and transmission setups. A total of 25 samples were used for training a regression model and cross-validation. The samples were measured in four series on two different days. As with the pure glucose spectra, the analysis was done on spectra with averaging over 255 pulses and ten spectra. Binning up to 510 pulses was also tested, but this did not yield significantly different RMSE-values in the regression analysis.

**Figure 5:**
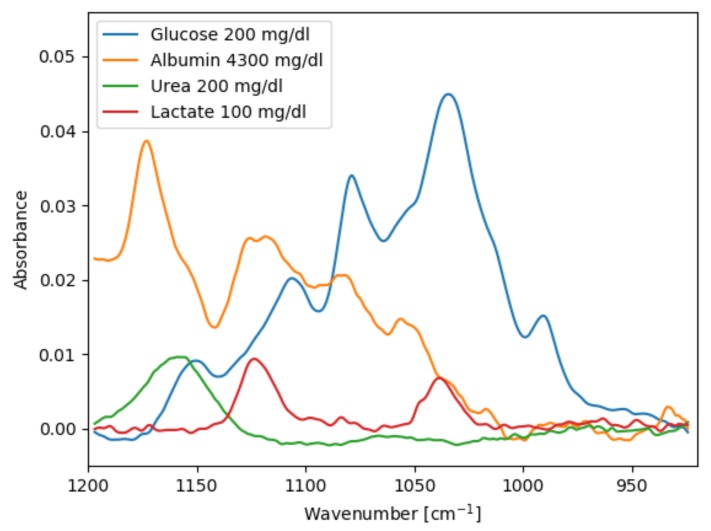
Absorbance spectra of pure analytes in demineralised water acquired in the transmission setup.

Figure 5 shows pure analyte spectra of the four species used in the samples, acquired in the transmission setup. Lactate has two absorption bands at 1040 cm${}^{-1}$ and 1124 cm${}^{-1}$ that overlap with glucose. Urea has only one small absorption band at approx. 1160 cm${}^{-1}$, but even this can interfere with glucose prediction depending on the latent variables found in the PLS model training. Albumin also has several absorption bands in the EC-QCL emission range. The concentration of albumin and other proteins is usually determined using the spectral region around 1600 cm${}^{-1}$, but it has been shown that the 1200–1000 cm${}^{-1}$ range is sufficient to determine total protein and albumin content in human blood plasma [37,38].

The normal physiological glucose concentration range for a healthy person is between 70 and 110 mg/dL. A blood glucose level (BGL) lower than 70 mg/dL is called hypoglycaemic, while a BGL above 180 mg/dL is called hyperglycaemic [39,40]. For people with diabetes the BGL can go up towards 400 mg/dL before hyperglycaemia symptoms become severe if the BGL goes unchecked. However, a broader glucose concentration range (0–800 mg/dL) has been used in these aqueous solutions in order to test the robustness of the measurement setups and the regression analysis. Similarly, concentration ranges of albumin, lactate, and urea (see Table 1) encompass human serum concentrations, but the highest concentrations used here are unlikely in physiological situations [38,41].

The prediction accuracies obtained for glucose and albumin are summarised in Table 3. In the ATR setup, an RMSECV of 16.3 mg/dL was achieved for glucose with L5OCV, while the RMSECV was 15.4 mg/dL in the transmission setup. The best prediction results were obtained using four or five latent variables. For cross-validation where one dataset was left out, the RMSECV for glucose was found to be 15.5 mg/dL for transmission measurements and 18.4 mg/dL for ATR measurements. This demonstrates that the setups were stable over time, as the prediction errors did not change significantly when datasets acquired at different times and on different days were used for cross-validation. The same trends were found for the analysis of albumin concentrations. For transmission spectroscopy, comparable prediction errors for glucose have been found in a previous study with a fiber-based EC-QCL setup [17], but this study used narrower concentration ranges, which can affect RMSE-values, and employed a reference arm for stability.

**Figure 6:**
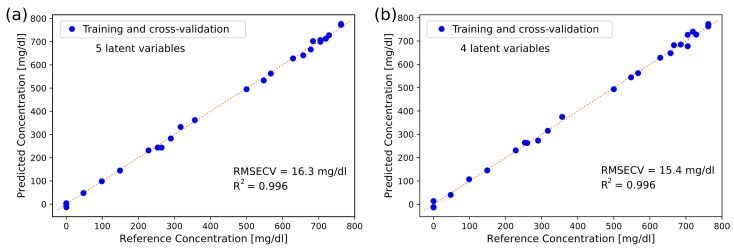
Glucose concentration levels in 25 aqueous samples plotted against reference concentrations with (**a**) the ATR setup for measurements and (**b**) the transmission setup for measurements. PLS regression with 5 or 4 latent variables and leave-5-out cross-validation was used for prediction.

Figure 6 shows the predicted glucose concentrations for L5OCV plotted against the reference concentrations for the measurements in the ATR and transmission setups. Glucose prediction is linear over the entire range, also at low physiological concentrations (<100 mg/dL). This, together with the RMSECV levels, indicates that our findings are significant for physiologically relevant glucose concentrations.

It was also found that the measurements in the transmission setup could be used to predict the concentrations of urea and lactate in the complex solutions. The RMSECV values for L5OCV were found to be 8.1 mg/dL for lactate and 9.9 mg/dL for urea. Accurate predictions could not be made for these analytes in the ATR setup (R${}^{2}<$ 0.5). This is likely due to a combination of a lower SNR in the ATR measurements and the overlap between lactate and urea absorption bands with the other analytes.

## 4. Discussion

An important feature of this system is the use of fiber-coupling, as fiber-coupling is very advantageous for further development of a practical glucose measurement system. Fiber-coupling enables simpler light guiding as compared to free-space, and light reflections from e.g., liquid cells are avoided. A fiber-based setup can also more easily be incorporated in miniaturised and portable sensors. Mid-infrared fibers have acceptable optical loss (approx. 0.3 dB/m) for these applications, although they are still quite expensive. The large-core fibers used in mid-infrared sensing are stiff and can be challenging to manipulate and align. Fiber-coupling also has the potential for more sources of loss, e.g., at the fiber-coupler. However, in our system the optical loss was <5% in the coupler and approximately 0.3 dB/m in the fibers, which was not significant for the results.

As expected when comparing measurements in ATR and transmission setups, the absorbance intensity was significantly lower in the ATR measurements due to the shorter interaction length. However, the RMSECV values for glucose and albumin measured in the ATR setup were still comparable to those from the transmission setup. This indicates that ATR measurements are sensitive enough for biomedical measurements despite the shorter interaction length. The geometry of an ATR fiber probe may also have several practical advantages over a transmission configuration. For example, a small transmission gap is prone to clogging and ensuring alignment of the fiber ends can be mechanically challenging. An ATR fiber probe is potentially more mechanically stable, since the measurand is on the surface of the prism rather than being in a gap between fibers. In addition, the input fiber can more easily also serve as an output fiber using an ATR probe.

The ZnS prism used for the ATR measurements was a suitable sensing interface, as the crystal surface was stable and easy to clean. ZnS is also well-suited in any potential in vivo applications, as it is non-toxic to humans. The main challenge with ATR spectroscopy and concentration predictions seemed to be the lower SNR as compared to the transmission measurements, which gave somewhat higher prediction errors. The primary cause behind this was the reduced interaction length. This effect may be ameliorated by further optimising the prism dimensions. For example, a prism with half the height (1.2 mm) would have twice as many internal reflections with approximately the same optical pathlength through the prism. Thus the interaction length with the sample could be increased, without increasing pathlength and material absorption.

The dimensions of the sensor should be reduced for portable sensing applications, and this can be achieved in several ways. One alternative is to create a fiber-coupled probe design with an ATR prism or looped bare optical fibers at the end of a fiber [5,42]. A fiber probe with an ATR prism necessitates a smaller crystal, down to single-reflection, with concomitant signal reduction. This signal reduction can be counteracted with the use of e.g., surface-enhanced infrared absorption spectroscopy (SEIRAS) [43]. SEIRAS can be achieved by coating the ATR prism with metal nanoparticles, but these substrates often have difficulties with stability and reproducibility. Another option is to fabricate micrometer structures onto the sampling facet of the ATR crystal. Such structures on ATR crystals have been shown to enhance the absorption signal up to a factor of 10–100 [44]. On the other hand, evanescent sensing with looped fibers can be used to maintain a long interaction length, while avoiding the need for surface engineering. However, looped optical fibers are fragile and would likely need to be encased in a flow-through chamber for stability.

For a portable sensor another avenue of size reduction is to use a smaller QCL source. As EC-QCLs are a recent commercial product, they will likely become somewhat smaller in the future. However, for personal and wearable sensors such as glucose sensors the dimensions must be reduced even further. One option is to use a few single-wavelength laser chips in order to target the most informative wavenumbers. It has been suggested several times that only a few wavenumbers are needed for an adequately accurate prediction of glucose concentrations, also for non-invasive measurements [27,41]. Robust measurements using a few wavenumbers must be tested thoroughly in realistic conditions in order to confirm this.

This study has demonstrated the use of an EC-QCL on samples with glucose and albumin as an example. However, we would like to emphasise that measurements with QCLs in applications for biomedicine and biotechnology have many additional potential uses where MIR spectroscopy with high sensitivity could replace other benchtop and portable measurement methods. In research, QCLs have so far also been used for breath analysis in order to detect disease markers, and in histopathology to e.g., detect cancer [2,45,46]. EC-QCLs also have many potential uses where they can outperform methods that today require labelling or reagents, such as protein analysis, cell studies and pharmaceutical and food analysis [47].

## 5. Conclusions

A fiber-coupled EC-QCL was used for multianalyte sensing in both ATR and transmission measurement configurations. Glucose and albumin concentrations were determined in solutions that also contained urea and lactate, with promising results. The RMSECV values for glucose were well under 20 mg/dL, even though a broad concentration range was used. The low cross-validation errors were also very promising considering the addition of other analytes with overlapping absorption bands. The simultaneous determination of several biomarkers is relevant for further sensor development, and the use of optimal design for analyte concentrations allowed for investigating the entire design space for four analytes with relatively few samples, while still obtaining robust analyte predictions. This is advantageous for testing and characterisation of sensor setups, as fewer total measurements are needed while precision is still maintained.

The intensity variation in the laser pulses was evaluated, and was decreased to an acceptable level with averaging while the measurement time did not exceed 10 s. Both setups were mechanically stable over time, and it was shown that measurements from different days could be used for concentration predictions. This concept study should be further assessed by measuring and analysing larger datasets, including samples of bodily fluids. As the ATR setup is fiber-coupled, it lends itself well to further developments towards a miniaturised and portable sensor.

Future developments include improving the SNR in the ATR measurements through optimisations of the prism dimensions. We will also investigate the feasibility of using a thin silicon crystal with micrometer surface structures for signal enhancement as an ATR element. This could be a viable alternative for minimally invasive or non-invasive fiber-probe sensors for on-demand sensing.

## Figures and Tables

**Table 1:** Concentration ranges of analytes used in the setups to test the measurement sensitivity for analytes with overlapping absorption bands.

Analytes	Concentration Range [mg/dL]
Glucose	0–800
Albumin	0–6000
Urea	0–200
Lactate	0–90

**Table 2:** Prediction errors for glucose in aqueous solutions obtained with PLS regression.

**ATR Measurements**			
**Cross-Validation**	**RMSECV [mg/dL]**	**R** ${}^{2}$	**LVs**
LOOCV	10.2	0.998	3
LDOCV	12.8	0.997	4
**Transmission Measurements**			
**Cross-Validation**	**RMSECV [mg/dL]**	**R** ${}^{2}$	**LVs**
LOOCV	7.5	0.999	3
LDOCV	9.7	0.998	4

**Table 3:** Concentration predictions for glucose and albumin in aqueous solutions measured in the ATR and transmission setups, using PLS regression.

**ATR Measurements**						
**Cross-Validation**	**Glucose**			**Albumin**		
	**RMSECV [mg/dL]**	**R** ${}^{2}$	**LVs**	**RMSECV [mg/dL]**	**R** ${}^{2}$	**LVs**
L5OCV	16.3	0.996	5	174.5	0.988	6
LDOCV	18.4	0.994	5	178.1	0.988	5
**Transmission Measurements**						
**Cross-Validation**	**Glucose**			**Albumin**		
	**RMSECV [mg/dL]**	**R** ${}^{2}$	**LVs**	**RMSECV [mg/dL]**	**R** ${}^{2}$	**LVs**
L5OCV	15.4	0.996	4	157.9	0.991	4
LDOCV	15.5	0.996	5	162.4	0.990	4
